# Effectiveness of Modified Ulnar Metaphyseal Wedge Osteotomy in Treating Ulnar Impaction Syndrome: A Comparative Clinical Study

**DOI:** 10.1007/s00423-026-04001-w

**Published:** 2026-03-02

**Authors:** Qiang Zhou, Xinlei Hu

**Affiliations:** 1 Department of Orthopedic Surgery, the Second Affiliated Hospital, Zhejiang University School of Medicine, Hangzhou, 310020 China; 2 Orthopedics Research Institute of Zhejiang University, Hangzhou City, Zhejiang Province China

**Keywords:** Retrospective study, Diaphyseal ulnar shortening osteotomy, Modified ulnar metaphyseal wedge osteotomy, Ulnar impaction syndrome

## Abstract

**Background:**

Ulnar shortening osteotomy (USO) is a standard surgical treatment for ulnar impaction syndrome. This study introduces a modified ulnar metaphyseal wedge osteotomy (MUMWO) and compares its outcomes with conventional diaphyseal ulnar shortening osteotomy (DUSO).

**Methods:**

In this retrospective cohort study, we compared postoperative functional outcomes and operative characteristics between patients undergoing traditional diaphyseal ulnar shortening osteotomy (n=18) and those receiving our modified ulnar metaphyseal wedge osteotomy (n=13) for treatment of ulnar impaction syndrome.

**Results:**

No differences were observed in patients characteriscs, ulnar variance, pain and functional score between two groups at the baseline. Patients underwent modified ulnar metaphyseal wedge osteotomy showed significant improvements in pain, Quick-DASH scores, wrist extension, wrist flexion, supination, and pronation (All *P*≤ 0.001). Compared to diaphyseal ulnar shortening osteotomy, modified ulnar metaphyseal wedge osteotomy was associated with less degree of ulnar shortening, shorter surgery time, and reduced intraoperative blood loss (All* P *< 0.05). After adequate follow-up time, modified ulnar metaphyseal wedge osteotomyshowed lower rate to remove implants, greater improvement in pain and Quick-Disability of the Arm, Shoulder, and Hand questionnaire scores (Both *P*<0.001).

**Conclusions:**

Modified ulnar metaphyseal wedge osteotomy of the ulnar shaft and epiphysis optimizes the osteotomy plane and angle, allowing for precise decompression while preserving sufficient bone mass. These biomechanical advantages contribute to better early functional recovery and reduced surgical trauma. However, its potential role in delaying DRUJ degeneration and other complications requires further validation through more large-scale, randomized clinical trials.

## Introduction

 Ulnar Impaction Syndrome (UIS) is a common degenerative wrist disorder that primarily affects individuals aged 20 to 50, with a slightly higher incidence in women. Occupational groups engaged in repetitive wrist movements, such as gymnasts and machine operators, are at significantly increased risk [1–4]. Research suggested that individuals with ulnar-positive variance have an incidence rate 3 to 5 times higher than the general population [1, 5, 6]. Clinically, UIS typically manifests as chronic, progressive pain on the ulnar side of the wrist, which worsens with pronation, supination, and gripping motions. Key clinical signs are as follows, tenderness on the ulnar aspect of the wrist joint, restricted range of motion, particularly in supination and ulnar deviation, reduced grip strength, localized swelling, possible clicking sounds during movement. Without timely intervention, UIS may progress to injuries of the triangular fibrocartilage complex (TFCC), further compromising quality of life [7, 8]. Multiple surgical techniques were reported, among which ulnar-shortening osteotomy (USO) was described as the reference standard for treating UIS [9, 10]. The most common surgery was diaphyseal ulnar shortening osteotomy (DUSO), and considerable short and mid-term effects were reported [11, 12]. However, many complications were demonstrated, including disorders of hardware removal [13–16], delay union or nonunion [17, 18], and complex pain syndrome [19, 20].

As an alternative osteotomy site, metaphyseal osteotomy has emerged as a promising approach for treating UIS. This technique offers several advantages, including a higher rate of bone healing, reduced need for specialized equipment, and a comparable reduction in load on the distal ulnocarpal joint [21–23]. However, controversies remain regarding the treatment of UIS, as the effectiveness of metaphyseal USO has not been fully explored, and reliable patient-reported outcomes are lacking. And despite numerous studies on this subject, there remain inconsistencies in the reported outcomes of the two methods [24–26]. Some studies suggest that one method is more effective in certain cases, while others indicate the opposite, with no consensus reached on the optimal approach for this patient populations.

This retrospective study aims to introduce a modified ulnar metaphyseal wedge osteotomy (MUMWO) and compare its radiographic and functional outcomes with those of diaphyseal osteotomy. Additionally, the study evaluates the surgical advantages and disadvantages associated with each technique.

## Materials and methods

After receiving approval from the Ethics Committee of the Second Affiliated Hospital of Zhejiang University School of Medicine, we retrospectively reviewed data from patients who underwent surgery for UIS at our hospital between 2020 and 2024. The inclusion and exclusion criteria were as follows: Adult patients aged 18 years or older who underwent primary USO after at least six months of failed nonsurgical treatment were included. UIS was diagnosed based on medical history and physical examination findings, such as wrist ulnar deviation and a positive ulnocarpal stress test. Radiographic confirmation was required, with positive ulnar variance observed on X-ray images taken with the forearm in a neutral position. Nonsurgical treatments used in this study included wrist immobilization, anti-inflammatory medications, and local steroid injections. Patients were excluded if they had degenerative joint diseases, osteoporosis (based on the Chinese guidelines for the diagnosis and treatment of primary osteoporosis) [27], a history of forearm or wrist fractures, prior surgery for congenital disorders, or an immature skeleton.

Patients were then grouped based on the type of surgery performed: DUSO or MUMWO. The selection process was not randomized, as the choice of surgical technique was made by the patients and their families after discussing the advantages and disadvantages of each approach with the surgical team.

Magnetic resonance imaging (MRI) was performed when UIS diagnosis is difficult. Patients whose TFCC was intact on MRI didn’t undergo wrist arthroscopy, while other patients were undergoing diagnostic wrist arthroscopy when surgery to confirm whether TFCC is intact. The presence of TFCC injury was recorded simultaneously during the procedure.

### Surgical techniques

#### Diaphyseal USO

After achieving successful brachial plexus anesthesia, the patient was positioned supine with the affected limb abducted. A pneumatic tourniquet was applied to the upper arm, and routine disinfection and draping were performed. Exsanguination was carried out, and the tourniquet pressure was set at 30 kPa.

**Fig. 1:**
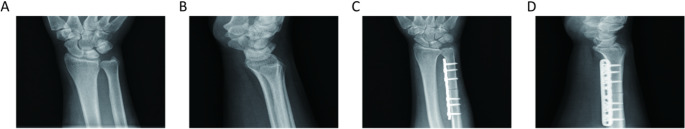
Preoperative and postoperative radiographs of DUSO. **A**-**B**. Preoperative radiographs of DUSO. C-D. Postoperative radiographs of DUSO. DUSO, diaphyseal ulnar shortening osteotomy

A longitudinal incision (~ 8 cm) was made at the junction of the middle and lower thirds of the ulnar forearm. The skin, subcutaneous tissue, and deep fascia were incised sequentially, taking care to protect the dorsal branch of the ulnar nerve. The interval between the flexor carpi ulnaris and extensor carpi ulnaris was dissected to expose the ulna, followed by subperiosteal dissection while preserving the periosteum. A 7-hole, 3.5 mm reconstruction plate (TriMed; UOCP, USA) was placed on the dorsal side of the ulna. A preliminary drill hole was made at the second distal hole of the plate, and a screw was inserted for temporary fixation. Two parallel osteotomy planes were marked between the third and fifth distal holes of the plate along the longitudinal axis of the ulna, with an interval approximately equal to the length of the positive ulnar variance. The distal screw was loosened, then plate was removed. Osteotomy was performed as planned, and the excised segment was removed. The ulna was then shortened and repositioned, ensuring tight alignment of the osteotomy surfaces. The plate was reattached, and sequential drilling, depth measurement, and screw insertion were performed proximally and distally to secure the osteotomy ends. Fluoroscopy with a C-arm X-ray machine was used in anteroposterior and lateral views to confirm complete correction of the positive ulnar variance, as well as proper alignment and apposition of the ulna. The tourniquet was released, and the wound was closed in layers. Preoperative and postoperative radiographs were shown in the Fig. 1.

### Modified ulnar metaphyseal wedge osteotomy

The preoperative preparation was similar with the DUSO. Then a curved incision, approximately 3–5 cm in length, was made along the ulnar border of the distal ulna, curving downward toward the distal radioulnar joint (DRUJ). The skin, fascia, and part of the extensor retinaculum were incised while taking care to protect the dorsal branch of the ulnar nerve. The extensor digiti minimi tendon and the extensor carpi ulnaris tendon were exposed and retracted laterally.

**Fig. 2:**
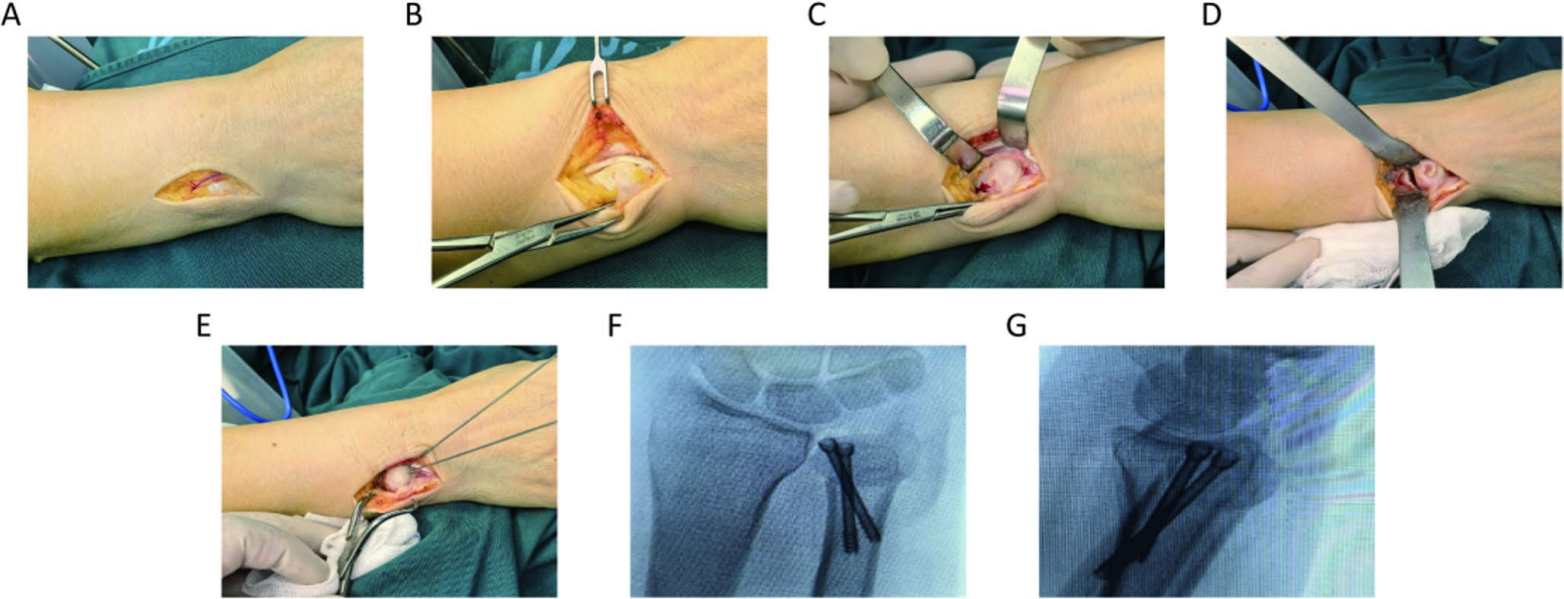
Sequential surgical steps of the MUMWO. (**A**) An L-shaped incision is made on the ulnar-dorsal aspect of the wrist. (**B**) The dorsal capsule is incised 2 mm dorsal to the extensor carpi ulnaris (ECU) tendon. (**C**) The dorsal ulnar head is exposed by reflecting the periosteum bilaterally. (**D**) Direct visualization of the osteotomy gap following wedge resection. (**E**) Temporary fixation is achieved using a reduction clamp and a dorsal K-wire. (**F**–**G**). Intraoperative views showing the final construct after definitive screw fixation

**Fig. 3:**
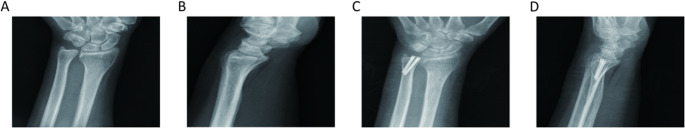
Preoperative and postoperative radiographs of MUMWO. **A**-**B**. Preoperative radiographs of MUMWO. **C-D**. Postoperative radiographs of MUMWO. MUMWO, modified ulnar metaphyseal wedge osteotomy

An inverted “L”-shaped capsular incision curving downward was made along the articular surface of the ulnar head to access the DRUJ. The proliferative and degenerated synovial tissue within the joint cavity was excised. A transverse osteotomy was performed at the proximal DRUJ, osteotomy length was determined by the ulnar variance, ensuring the integrity of the ulnar-side cortical bone of the ulna. A second oblique osteotomy was then made 3–5 mm proximal to the first osteotomy, with the two osteotomy lines intersecting at the radial border of the ulna while preserving the continuity of the radial cortex and periosteum. Bone forceps were used to clamp both osteotomy ends, applying downward pressure to depress the distal osteotomy fragment. Two headless compression screws guidewires were inserted obliquely through the osteotomy site, penetrating the proximal cortical bone. Fluoroscopic confirmation with a C-arm X-ray machine ensured proper guidewire placement and adequate distal fragment depression. The two headless compression screws (Synthes, GmbH, PA, USA) were then inserted along the guidewires for stable fixation (Fig. 2). The joint capsule was repaired in a neutral position, followed by closure of the extensor retinaculum and skin in layers. Preoperative and postoperative radiographs were shown in the Fig. 3.

In patients diagnosed with TFCC injury in both groups, a TFCC repair surgery was performed when combined with unstable distal radioulnar joint. A transverse incision was made in the distal portion of the TFCC joint capsule to explore the TFCC. Degenerated synovial tissue was debrided. If a TFCC tear was present, it was repaired using a 3 − 0 PDS suture with a dorsal capsular technique. If the TFCC was avulsed from the fovea of the ulna, reconstruction was performed either arthroscopically via bone tunnel reconstruction or with suture anchors.

In both groups, postoperative management includes routine immobilization with a long-arm orthosis for three weeks, followed by a short-arm orthosis for continued fixation. Functional exercises are initiated under non-weight-bearing conditions. Weight-bearing activities are permitted once X-ray examination confirms bone callus formation. Postoperative follow-ups are scheduled at 3 weeks, 6 weeks, 3 months, 6 months, and 12 months.

### Data collection

The follow-up period was calculated from the date of surgery to the date of the last review. Data on age, sex, height, weight, smoking status, ulnar variance, ulnar shortening amount, operation time, blood loss volume, and type of instrumentation were collected from electronic medical records. Pain was assessed using the Visual Analogue Scale (VAS) [28, 29], while forearm function was evaluated with the Quick-Disability of the Arm, Shoulder, and Hand (Quick-DASH) score [30, 31]. Wrist range of motion was recorded and analyzed. Additionally, complications associated with each surgical technique and the need for implant removal were documented. Bone healing was assessed via radiographic evaluation. The healing time was recorded when radiographs demonstrated bridging callus formation with obscured fracture lines.

### Data analyses

After data collection, the normality of the distribution was assessed using the Shapiro–Wilk W test. Continuous variables were presented as mean ± standard deviation (SD), while categorical variables were expressed as counts (percentages). A Kruskal Wallis test was used to analyze continuous variables, and a chi-square test was applied for categorical variables. All statistical analyses were conducted using Stata version 17.0 (Stata Corp, Texas, United States). A two-sided p-value < 0.05 was considered statistically significant.

## Results

A total of 31 patients were enrolled in the study, including 18 who underwent DUSO and 13 who underwent MUMWO. Most cases were attributed to isolated positive ulnar variance, with a similar distribution of causes between the two groups. No significant differences were observed between the groups in terms of age, gender, body mass index, smoking status, preoperative ulnar variance, TFCC injury, or DRUJ patterns (All *P* > 0.05) (Table 1).

**Table 1:** Patient characteristics and outcomes for Diaphyseal Ulnar Shortening Osteotomy (DUSO) versus modified ulnar metaphyseal wedge osteotomy (MUMWO)

Patient characteristics	DUSO	MUMWO	*P*-value
Number	18	13	
Age, y	47.06 ± 11.78	53.92 ± 10.73	0.113
Gender (% of female)	44.44%	76.92%	0.071
Body mass index, kg/m^2^	23.87 ± 3.84	24.04 ± 4.25	0.968
Active smokers (%)	23.08%	22.22%	0.955
Ulnar varience, mm	3.86 ± 2.27	3.14 ± 1.48	0.614
TFCC injury	72.22%	69.23%	0.305
DRUJ patterns			0.995
parallel	61.11%	61.54%	
oblique	22.22%	23.08%	
reverse oblique	16.67%	15.38%	

*y* years old, *kg* kilogram, *m*^*2*^ square of meters, *mm* micromillimeter

After an adequate follow-up period of 18.54 ± 7.45 months, patients who underwent MUMWO surgery showed significant improvements in pain, Quick-DASH scores, wrist extension, wrist flexion, supination, and pronation (All *P* ≤ 0.001) (Table 2). Compare to the DUSO group, MUMWO group was associated with a lower Quick DASH score and a shorter bone healing time (Both *P* < 0.001). No significant differences were observed in follow-up time, pain, wrist extension, wrist flexion, supination, and pronation in both groups (All *P* > 0.05) (Table 3). The MUMWO group had a less degree of ulnar shortening compared to the DUSO group (*P* = 0.035). Furthermore, the MUMWO procedure was associated with a shorter operative time, reduced intraoperative blood loss, and a lower rate of implant removal postoperatively (All *P* < 0.001). However, the percentage of patients requiring additional TFCC repair was similar between the two groups (Table 4).

**Table 2:** Pain and function parameter outcomes for MUMWO

Function variables	Preoperative	Postoperative	*P*-value
Wrist flexion	52.46 ± 5.74	63.46 ± 4.24	**< 0.001**
Wrist extension	57.08 ± 4.09	74.85 ± 3.93	**< 0.001**
Pronation	73.31 ± 6.13	81.85 ± 5.87	**0.001**
Supination	60.54 ± 5.44	81.92 ± 5.17	**< 0.001**
Pain, VAS score	6.46 ± 1.61	2.69 ± 0.85	**< 0.001**
Quick DASH score	66.59 ± 6.38	20.80 ± 6.21	**< 0.001**

*VAS* Visual Analogue Scale, *Quick-DASH* Quick-Disability of the Arm, Shoulder, and Hand

**Table 3:** Preoperative and Postoperative wrist function for two operation techniques

Function variables	DUSO	MUMWO	*P*-value
Follow-up time, mon	24.06 ± 9.39	18.54 ± 7.45	0.063
Pain, VAS score	Preoperative: 6.50 ± 1.50	Preoperative:6.46 ± 1.61	0.984
	Postoperative: 3.00 ± 1.37	Postoperative: 2.69 ± 0.85	0.676
Quick DASH score	Preoperative: 62.87 ± 5.25	Preoperative: 66.59 ± 6.38	0.078
	Postoperative:40.85 ± 6.63	Postoperative: 20.80 ± 6.21	**< 0.001**
Wrist flexion	Preoperative: 54.78 ± 4.78	Preoperative: 52.46 ± 5.74	0.206
	Postoperative:62.67 ± 4.06	Postoperative:63.46 ± 4.24	0.643
Wrist extension	Preoperative: 55.39 ± 4.58	Preoperative: 57.08 ± 4.09	0.507
	Postoperative: 75.94 ± 5.40	Postoperative: 74.85 ± 3.93	0.616
Pronation	Preoperative: 72.11 ± 4.00	Preoperative: 73.31 ± 6.13	0.574
	Postoperative: 78.39 ± 7.39	Postoperative: 81.85 ± 5.87	0.253
Supination	Preoperative: 59.39 ± 6.56	Preoperative: 60.54 ± 5.44	0.520
	Postoperative: 78.39 ± 7.20	Postoperative: 81.92 ± 5.17	0.118
Bone healing, mon	7.06 ± 3.35	3.46 ± 1.45	**< 0.001**

*mon* month, *VAS* Visual Analogue Scale, *Quick-DASH* Quick-Disability of the Arm, Shoulder, and Hand

**Table 4:** Surgical variables for two operation techniques

Variables	DUSO	MUMWO	*P*-value
TFCC repair	5/18(27.78%)	4/13(30.76%)	0.305
Shortening, mm	4.61 ± 2.39	2.98 ± 1.06	**0.035**
Operation time, min	82.50 ± 25.97	53.85 ± 15.96	**< 0.001**
Volume of bleeding, ml	21.39 ± 22.15	5.23 ± 4.66	**< 0.001**
Implant removal, %	38.89%	0%	**< 0.001**

*mm* micromillimeter, *ml* milliliter

A total of patients 22(70.97%) were diagnosed with TFCC injury and 9(29.03%) patients subsequently underwent additional TFCC repair surgery. Compared to patients with an intact TFCC who did not receive repairing, those who underwent TFCC repair showed similar outcomes in terms of pain, Quick-DASH score, wrist flexion, wrist extension, pronation, and supination in the later stages of recovery (Table 5).

**Table 5:** Preoperative and Postoperative wrist function according to the status TFCC repair

Function variables	No repair	TFCC repair	*P*-value
Pain, VAS score	Preoperative: 6.46 ± 1.56	Preoperative:6.22 ± 1.72	0.731
	Postoperative: 3.00 ± 1.22	Postoperative: 2.33 ± 0.71	0.206
Quick DASH score	Preoperative: 64.33 ± 6.07	Preoperative: 64.10 ± 6.42	0.687
	Postoperative: 32.00 ± 14.40	Postoperative: 34.98 ± 9.35	0.947
Wrist flexion	Preoperative: 53.92 ± 5.81	Preoperative:54.44 ± 6.35	0.313
	Postoperative:63.00 ± 4.55	Postoperative:63.22 ± 4.49	0.524
Wrist extension	Preoperative: 56.85 ± 4.18	Preoperative: 54.67 ± 4.66	0.568
	Postoperative: 76.46 ± 5.64	Postoperative: 74.67 ± 4.69	0.592
Pronation	Preoperative: 72.08 ± 5.17	Preoperative: 73.11 ± 5.62	0.482
	Postoperative: 80.85 ± 5.73	Postoperative: 82.11 ± 5.51	0.920
Supination	Preoperative: 60.92 ± 6.87	Preoperative: 58.78 ± 4.94	0.920
	Postoperative: 78.77 ± 5.43	Postoperative: 79.22 ± 8.17	0.526

*VAS* Visual Analogue Scale, *Quick-DASH* Quick-Disability of the Arm, Shoulder, and Hand

Few complications were observed. Two patients who underwent DUSO developed DRUJ arthritis during follow-up. One patient required bone grafting due to nonunion who underwent DUSO, though eventual healing was achieved. No cases of nonunion or malunion were observed with MUMWO technique. No wound-related complications occurred, and all patients exhibited complete bone union by the final follow-up.

## Discussion

The primary goal of surgical treatment for UIS is to correct ulnar positive variance and restore biomechanical balance in the wrist joint. This study compared the clinical outcomes of DUSO and MUMWO, demonstrating that MUMWO offers significant advantages in functional recovery efficiency, surgical safety, and postoperative complication management.

Our findings indicated that the degree of ulnar shortening in the MUMWO group was significantly less than in the DUSO group. Anatomical studies suggest that the metaphyseal trabecular structure has a higher elastic modulus, allowing for precise regulation of the distal ulnar joint surface angle through wedge osteotomy [32, 33]. Unlike DUSO, which primarily shortens the ulna, MUMWO may better accommodate the biomechanical demands of dynamic wrist loading by modifying the inclination angle of the distal ulnar joint surface [34]. Additionally, the metaphysis has a rich blood supply, mainly from the periosteal vascular network and nutrient artery branches, which accelerates bone healing by approximately 30% compared to the diaphysis [35, 36]. Furthermore, the MUMWO technique preserves the integrity of the ulnar cortical bone. These could explain why secondary bone grafting was unnecessary in the MUMWO group and why the implant removal rate was lower.

Although no statistically significant difference was observed between the two groups in terms of final wrist range of motion (flexion/extension/pronation/supination), the MUMWO group showed greater improvement in functional scores (Quick-DASH). This may be attributed to the modified surgical technique’s optimized adjustment of the DRUJ rotation axis, which reduces persistent stress on the TFCC. Biomechanical studies support this hypothesis, showing that when the osteotomy plane is closer to the DRUJ, each millimeter of osteotomy adjustment can reduce joint contact pressure [37, 38]. In the DUSO group, two cases of DRUJ arthritis were observed during follow-up, whereas no such complication occurred in the MUMWO group. These findings suggest that MUMWO may have less short-term impact on DRUJ integrity, though longer-term effects require further investigation.

The MUMWO group also demonstrated advantages in operative time and intraoperative blood loss, reflecting adherence to minimally invasive surgical principles. Metaphyseal osteotomy does not require extensive periosteal or soft tissue stripping, reducing the risk of injury to the dorsal branch of the ulnar nerve while preserving the anatomical attachment points of the pronator quadratus muscle. This is particularly important for early postoperative rehabilitation, as the integrity of pronator quadratus function directly influences the speed of forearm rotation recovery.

In this study, 70.96% of cases were diagnosed with TFCC injury. However, only 29.03% of cases required concurrent TFCC repair, aligning with the reported incidence of type II-IV injuries in Palmer’s classification (25–35%) [39, 40]. Notably, there was no significant difference in final functional scores between the TFCC repair group and the non-repair group. While this finding may be influenced by the limited sample size, it also suggests that for stable TFCC injuries—such as central perforations without DRUJ instability—simple osteotomy may be sufficient to alleviate symptoms. This supports the “osteotomy-first” strategy recommended in the latest guidelines, which prioritize correcting osseous abnormalities when ulnar positive variance exceeds 2.5 mm, with soft tissue repair decisions made based on intraoperative arthroscopic findings [10, 41]. However, any extrapolation of these results should be approached with caution, as initially stable TFCC injuries may develop delayed instability post-surgery due to biomechanical changes. A more refined TFCC classification system and extended follow-up are necessary to assess long-term complications and outcomes.

Although MUMWO offers several advantages, there are still some limitations to consider. One potential limitation is the baseline characteristic bias between the two groups. While statistical analysis showed no significant differences, the DUSO group had a larger sample size (18 vs. 13), which may affect statistical power. Additionally, the follow-up period may not be sufficient to assess late complications following osteotomy. Expanding the sample size and extending the follow-up duration are necessary to further validate the clinical significance of functional outcomes. Moreover, this study was conducted at a single center and was not randomized, which may limit the generalizability of the findings. Further large-scale, randomized clinical trials are warranted to comprehensively evaluate the long-term outcomes of these surgical techniques. What’s more, though this technique showed promised effect in the present manuscript, one potential limitation of our technique, metaphyseal wedge osteotomy, is its reduced effectiveness in cases where ulnar styloid impaction is the primary pathology. We acknowledge that in such cases, the impaction of the ulnar styloid may not be sufficiently addressed by the osteotomy alone. Our technique is primarily designed for patients who present with ulnar-sided wrist pain primarily due to ulnar variance, rather than isolated styloid impaction. This distinction is important as the pathological mechanisms differ, and our approach is tailored to address the specific issues related to ulnar variance. In instances where ulnar styloid impaction is the dominant issue, alternative surgical approaches, such as direct styloid resection or other procedures targeting the styloid process, may provide more effective outcomes. Further research and clinical studies are needed to explore the specific efficacy of metaphyseal wedge osteotomy in such cases, and whether combining it with additional treatments for styloid impaction could improve results.

In conclusion, the improved wedge-shaped osteotomy of the ulna shaft and epiphysis optimizes the osteotomy plane and angle, allowing for precise decompression while preserving sufficient bone mass. These biomechanical advantages contribute to better early functional recovery and reduced surgical trauma. This study provides new evidence-based support for the selection of surgical techniques for UIS; however, its potential role in delaying DRUJ degeneration and other complications requires further validation through long-term follow-up.

## Data Availability

The datasets used and/or analyzed during the current study are available fromthe corresponding author on reasonable request.
